# The impact of government reimbursement negotiation on targeted anticancer medicines use and cost in China: A cohort study based on national health insurance data

**DOI:** 10.7189/jogh.13.04083

**Published:** 2023-08-11

**Authors:** Yu Yang, Yichen Zhang, Anita K Wagner, Huangqianyu Li, Luwen Shi, Xiaodong Guan

**Affiliations:** 1 Department of Pharmacy Administration and Clinical Pharmacy, School of Pharmaceutical Sciences, Peking University, Beijing, China; 2 Department of Population Medicine, Harvard Medical School and Harvard Pilgrim Health Care Institute, Boston, Massachusetts, USA; 3 International Research Center for Medicinal Administration, Peking University, Beijing, China

## Abstract

**Background:**

High prices of targeted anticancer medicines (TAMs) result in financial toxicity for patients and the health insurance system. How national price negotiation and reimbursement policy affect the accessibility of TAMs for cancer patients remains unknown.

**Methods:**

In this population-based cohort study, we used national health insurance claims data in 2017 and identified adult patients with cancer diagnoses for which price-negotiated TAMs were indicated. We estimated the half-month prevalence of price-negotiated TAMs use before and after the policy implementation in September 2017. We calculated direct medical costs, out-of-pocket (OOP) costs, and the proportion of OOP cost for each cancer patient to measure their financial burden attributable to TAMs use. We performed segmented linear and multivariable logistic regression to analyse the policy impact.

**Results:**

We included 39 391 of a total 118 655 cancer beneficiaries. After September 2017, the prevalence of price-negotiated TAMs use increased from 1.4%-2.1% to 2.9%-3.1% (*P* = 0.005); TAMs users’ daily medical costs increased from US$261.3 to US$292.5 (*P* < 0.001), while median daily OOP costs (US$68.2 vs US$65.7; *P* = 0.134) and OOP costs as a proportion of daily medical costs persisted (28.5% vs 28.5%; *P* = 0.995). Compared with resident beneficiaries, the relative probability of urban employee beneficiaries on TAMs uses decreased after the policy (adjusted odds ratio (aOR) = 2.4 vs aOR = 2.2).

**Conclusions:**

The government price negotiation and reimbursement policy improved patient access to TAMs and narrowed disparities among insurance schemes. China’s approach to promoting the affordability of expensive medicines provides valuable experience for health policy decision-makers.

Cancer is a leading cause of death globally [1]. With advancements in precision oncology, some targeted anticancer medicines (TAMs) have improved the survival and quality of life of patients suffering from previously hard-to-treat cancers [2-4], but their high cost limits their use and possibly their effect on cancer patients’ mortality [5]. This has caused high concern among health care professionals [6]. In high-income countries such as the United States, targeted therapies dominate anticancer drug spending [7], with median total targeted drug costs per treatment estimated to be US$123 072 higher than traditional chemotherapy costs [8]. Moreover, rising prices of TAMs threaten even insured patients’ financial status [9]. Unresolved financial hardship may lead to increased risk of suboptimal treatment, treatment drop-out, symptom burden, and premature death [10,11]. In China, chemotherapy and cetuximab- or bevacizumab-combined therapy is recommended by guidelines as first- or second-line treatment of metastatic colorectal cancer, yet less than a fifth of patients receive it due to its high costs. The mean direct medical cost per cycle for patients who used combined chemotherapy and TAMs was US$931.1 higher than for those who used chemotherapy alone [12].

China has been implementing many policies to promote health care access, including access to anticancer medicines since 2009. As insurance coverage is a prerequisite for access to expensive anticancer medicines, adding them to China's National Reimbursement Drug List (NRDL), which guides health insurance reimbursement across the country, is key in enhancing their affordability and availability for patients [13]. China’s Basic Medical Insurance (BMI) schemes enrolled more than 95% of Chinese citizens by the end of 2018 [14], while additional insurances for critical illness and medical assistance are available to protect vulnerable populations. These insurance schemes work as a whole insurance system that protects Chinese residents from medical-related expenditures [15]. To decrease the financial burden and improve cancer patients’ access to effective therapy, the government sought to control prices or reduce out-of-pocket (OOP) costs of expensive TAMs [16]. Studies have confirmed that centralised negotiation or other regulatory approaches are effective in controlling medicine prices [17-20]. Besides, expanding the coverage of universal health insurance to reimburse expensive medicines can decrease burden of high OOP costs and could improve population health [21,22]. Similar to other countries, China has been implementing price negotiation of drugs since 2017 to increase insurance coverage while containing spending. After the 2017 national price negotiation, 15 TAMs were added to the NRDL, with negotiated prices reduced by 44% on average [23]. Following NRDL inclusion, provinces are required to update their Provincial Reimbursement Drug Lists. Public hospitals must then purchase these medicines through the provincial procurement websites, at no higher than the negotiated prices. Then, medicine expenses must be reimbursed by city- or county-level health insurance fund in accordance with the negotiated prices (minus beneficiaries’ co-payments) [24].

Previously, we have shown that the procurement volume of the 15 price-negotiated TAMs increased by 143.0%, while costs per defined daily dose decreased by 48.9% at six months after implementing the 2017 NRDL expansion [24]. However, as benefit packages vary across insurance schemes and regions in China, people were subject to different levels of financial protection covered by the health insurance schemes across different provinces, resulting in inequity [25,26]. We hypothesised that the expansion of NRDL would have reduced medical costs and inequality at the individual level. In this population-based cohort study, we aim to explore changes in the prevalence of use and direct medical costs of the 15 price-negotiated TAMs before and after the implementation of the NRDL expansion in September 2017 and to compare the probability of their use among patients with different socioeconomic characteristics.

## METHODS

### Data source

We used the 2017 nationwide database from the China Health Insurance Research Association (CHIRA), which conducted a two-stage sampling design to collect BMI beneficiaries’ claim data. Since 2015, the BMI consists of two health insurance schemes: the Urban Employee Basic Medical Insurance (UEBMI) for urban and retired employees and the Urban and Rural Resident Medical Insurance (URRMI) for other residents [13]. We used systematic random sampling to extract 2% of the UEBMI and URRMI beneficiaries residing in municipalities and provincial capitals, 5% of those in prefecture-level cities, and 10% of those in counties [27]. We extracted medical records in the sampling year for each sampled beneficiary.

The CHIRA database contains annual records of services received by a nationally representative sample of beneficiaries and has been widely used in studies in China [28,29], The database collects information of each visits on patient demographics (e.g. sex, age, and region), medical admission type (i.e. inpatient, outpatient, and pharmacy visit), diagnoses, health care service utilisation, medicines prescribed, and detailed health care expenditures and insurance payments for all services beneficiaries received [30]. Since the database was anonymised and de-identified, we did not require ethics approval.

### Sample medicines and study population

We included 15 TAMs that were added to the NRDL after the 2017 price negotiation, during which no generic medicines were available on the market (Table S1 in the **Online Supplementary Document**). Using the International Classification of Diseases 10^th^ Revision (ICD-10) code and indications stipulated by NRDL, we identified populations diagnosed with at least one of the 13 matched cancer types (C11 – Malignant neoplasm of nasopharynx, C16 – Malignant neoplasm of stomach, C18 – Malignant neoplasm of colon, C20 – Malignant neoplasm of rectum, C22 – Malignant neoplasm of liver and intrahepatic bile ducts, C25 – Malignant neoplasm of pancreas, C34 – Malignant neoplasm of bronchus and lung, C50 – Malignant neoplasm of breast, C64 – Malignant neoplasm of kidney, except renal pelvis, C83- Non-follicular lymphoma, C84 – Mature T/NK-cell lymphomas, C85 – Other and unspecified types of non-Hodgkin lymphoma, and C90 – Multiple myeloma and malignant plasma cell neoplasms), associated with the 15 price-negotiated TAMs from 1 January to 31 December 2017. We excluded patients without inpatient records and those <18 years old since none of these TAMs have paediatric indications. We extracted all medical records of sample cancer patients in 2017 for data analysis (Figure S1 in the **Online Supplementary Document**).

### Outcome measures

We used the prevalence change of TAMs use and total direct medical costs per patient to assess the impact of the 2017 NRDL expansion. We identified TAMs users as cancer patients who had been prescribed sample price-negotiated medicines during a specific period. We calculated prevalence by dividing the number of users by the total number of patients in the same half-month period. Direct medical costs contained costs of medicines, surgery, examinations, care, and other medical management, which consist of both reimbursement payments and OOP costs. Cost measures in our analysis included daily medical cost, daily OOP cost, and proportion of OOP expenditure. We defined daily medical costs as the individual direct medical costs divided by the length of stay during admission month. Similarly, we calculated daily OOP costs during the month of admission, with the individual OOP costs divided by the length of stay. We defined the proportion of OOP expenditure as the ratio of individual OOP to direct medical costs. Costs were converted into and reported in $US based on the average exchange rate in 2017 (US$1 = 6.75CNY) [31].

Additionally, we chose odd ratios (OR) of users as an outcome measure indicating disparities among patients with different socioeconomic characteristics (i.e. participating in different social health insurance schemes or living in different regions), considering the uneven levels of development in various regions and the variations in policy deductibles and levels of coinsurance between the insurance schemes.

### Statistical analysis

We conducted descriptive analyses of sample characteristics, the prevalence of TAM use, and direct medical costs before and after September 2017 – the time point of policy implementation considering the lag effect. To evaluate changes in the trend of the half-monthly prevalence of price-negotiated TAMs use among sample patients after September 2017, we performed a quasi-experimental interrupted time series analysis with segmented linear regression [32]. We then applied a multivariable logistic regression model to calculate adjusted odd ratios (aOR) with sex, age, health insurance scheme, region, and cancer site as control variables. We categorised the regions into eastern (11 relatively developed provinces), central (eight less developed provinces), and western (12 developing provinces), as per the China Health Statistic Yearbook [31]. We illustrated the medians and interquartile ranges (IQRs) of the three cost measures, comparing the former to determine whether there was a significant difference before and after September 2017. We performed all statistical analyses in STATA 15 (StataCorp, College Station, TX, USA).

## RESULTS

### Characteristics

We included 39 391 out of 118 655 BMI beneficiaries with a cancer diagnosis identified in the CHIRA database in 2017. The patients were mostly male (n = 20 967 (53.2%)), ≥65 years old (n = 22 255 (56.5%)), and URRMI beneficiaries (n = 25 099 (63.7%)). Eastern, central, and western regions contributed 31.0%, 45.1%, and 23.9% of the study population, respectively (**Table 1**). The most common cancer diagnoses were bronchus and lung cancer (n = 11 241 (28.5%)), breast cancer (n = 7606 (19.3%)), and stomach cancer (n = 4766 (12.1%)).

**Table 1:** Characteristics of patients included in analysis (n = 39 391)

Characteristics	n (%) of patients
**Sex**	
Male	20 967 (53.2)
Female	18 424 (46.8)
Age in years*	
18-44	3432 (8.7)
45-60	13 704 (34.8)
>60	22 255 (56.5)
**Health insurance scheme**	
URRMI	14 292 (36.3)
UEBMI	25 099 (63.7)
**Region†**	
Eastern	12 212 (31.0)
Central	17 746 (45.1)
Western	9433 (23.9)
**Cancer site‡**	
C11 Nasopharynx	1201 (3.0)
C16 Stomach	4766 (12.1)
C18 Colon	3729 (9.5)
C20 Rectum	3344 (8.5)
C22 Liver and intrahepatic bile ducts	3605 (9.2)
C25 Pancreas	891 (2.3)
C34 Bronchus and lung	11 241 (28.5)
C50 Breast	7606 (19.3)
C64 Kidney, except renal pelvis	825 (2.1)
C83 Non-follicular lymphoma	87 (0.2)
C84 Mature T/NK-cell lymphomas	78 (0.2)
C85 Other and unspecified types of non-Hodgkin lymphoma	1441 (3.7)
C90 Multiple myeloma and malignant plasma cell neoplasms	577 (1.5)

URRMI – Urban and Rural Resident Basic Medical Insurance, UEBMI – Urban Employee Basic Medical Insurance.

*Patients were categorised into young adults (18-44 years), middle-aged adults (45-59 years), and the old (>60 years) [33].

†Regions were classified by geographic areas (Eastern, Central, and Western) as per the National Bureau of Statistics [31]. Eastern region: Beijing, Tianjin, Hebei, Liaoning, Shanghai, Jiangsu, Zhejiang, Fujian, Shandong, Guangdong, and Hainan; Central region: Shanxi, Jilin, Heilongjiang, Anhui, Jiangxi, Henan, Hubei, and Hunan; Western region: Inner Mongolia, Chongqing, Guangxi, Sichuan, Guizhou, Yunnan, Xizang, Shaanxi, Gansu, Qinghai, Ningxia, and Xinjiang.

‡Cancer sites were classified according to the International Classification of Diseases 10^th^ Revision (ICD-10) code.

### Prevalence of price-negotiated TAMs use

**Figure 1:**
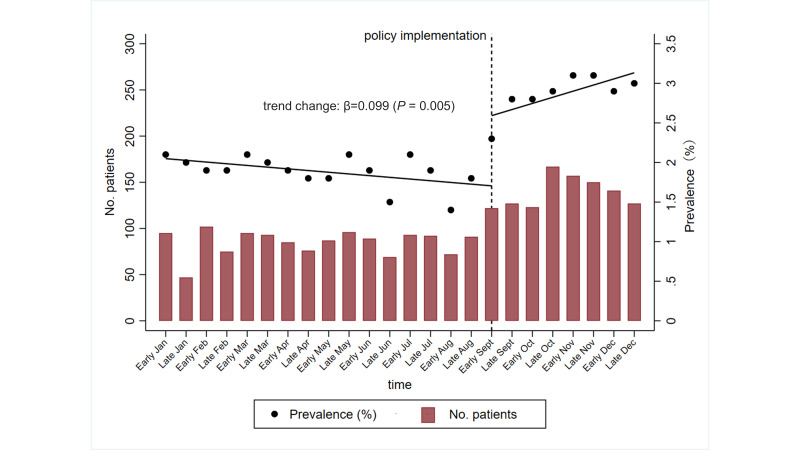
Trend in prevalence of price-negotiated TAMs use in 2017. The trend change of half-monthly prevalence of TAMs use was assessed by a quasi-experimental interrupted time series analysis with segmented linear regression.

The prevalence of TAMs use every half month ranged from 1.4% to 2.1% before September 2017 and from 2.9% to 3.1% after implementation (**Figure 1**). Implementation of the 2017 price negotiation policy was associated with a significant positive increase in the trend of half-monthly prevalence (correlation coefficient (β) = 0.099; 95% confidence interval (CI) = 0.034-0.164, *P* = 0.005).

**Figure 2:**
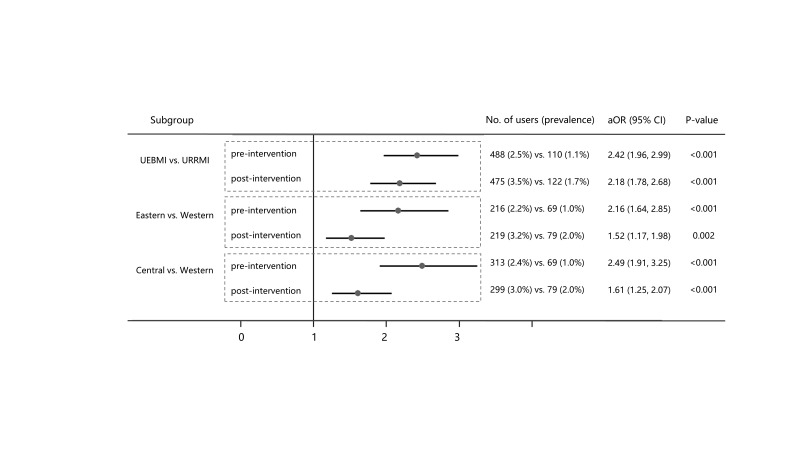
Multivariable logistic regression analysis of patients in different Basic Medical Insurance schemes or regions. *Multivariable logistic regression includes sex, age, health insurance scheme, region, and cancer site. aOR – adjusted odd ratio, URRMI –Urban and Rural Resident Basic Medical Insurance, UEBMI –Urban Employee Basic Medical Insurance.

We examined the probability of being prescribed price-negotiated TAMs among patients in different insurance schemes or geographical regions before and after September 2017 (**Figure 2**). Compared with URRMI beneficiaries, the aOR of UEBMI beneficiaries decreased from 2.42 (95% CI = 1.96-2.99) to 2.18 (95% CI = 1.78-2.68) after policy implementation. Compared to patients in the western region, the aOR of patients in the eastern (aOR = 2.16; 95% CI = 1.64-2.85 vs aOR = 1.52; 95% CI = 1.17-1.98) and central regions (aOR = 2.49; 95% CI = 1.91-3.25 vs aOR = 1.61; 95% CI = 1.25-2.07) decreased significantly.

### Costs of price-negotiated TAMs

Among sample patients, median direct daily medical costs were $165.6 and US$164.1 before and after September 2017, with a statistically non-significant absolute reduction of US$1.5 (*P* = 0.103, **Table 2**). Additionally, the median daily OOP costs and the proportion of OOP costs significantly declined from US$41.7 to US$37.7 and from 29.1% to 27.2%, respectively.

**Table 2:** Direct medical costs (US$) of sample patients*

	Full sample†			TAMs users‡		
**Expenditure type**	**Pre-intervention**	**Post-intervention**	***P*-value§**	**Pre-intervention**	**Post-intervention**	***P*-value§**
Daily medical costs	165.6 (103.1-269.2)	164.1 (101.1-270.8)	0.103	261.3 (158.0-492.3)	292.5 (167.5-520.4)	<0.001
Daily out-of-pocket costs	41.7 (18.0-84.7)	37.7 (15.4-81.2)	<0.001	68.2 (30.4-180.0)	65.7 (27.1-153.8)	0.134
Proportion of out-of-pocket costs	29.1 (15.7-44.0)	27.2 (14.2-41.5)	<0.001	28.5 (17.5-45.2)	28.5 (14.7-40.6)	0.995

TAM – targeted anticancer medicine, IQR – interquartile range

*Values presented as median (IQR) unless otherwise specified.

†Full sample included all the patients included in the analysis (n = 39 391).

‡TAMs users included patients who had been prescribed price-negotiated TAMs at least once in 2017 (n = 1017).

§Group differences were tested using median test. Values with *P* < 0.05 are considered statistically significant.

Among users prescribed with price-negotiated TAMs at least once in 2017, there was an increase in daily medical costs (US$261.3 vs US$292.5; *P* < 0.001) after policy implementation. There was no statistically significant difference in daily OOP costs (US$68.2 vs US$65.7; *P* = 0.134) and the proportion of OOP expenditure among TAM users (28.5% vs 28.5%; *P* = 0.995) (**Table 2**).

## DISCUSSION

The implementation of the price negotiation and reimbursement policy significantly increased the prevalence of price-negotiated TAMs use without raising users’ OOP burden. We also observed that differences in the prescribing rate of price-negotiated TAMs decreased among patients who participated in different insurance schemes or lived in regions with various economic levels. This means that the policy improved accessibility to TAMs and promoted health equity among cancer patients across mainland China.

Medical insurance is a major protective factor for healthcare utilisation [34]. A previous study found that, after price negotiation without mandatory reimbursement of three anticancer medicines, the hospital purchasing volume increased while procurement spending decreased [19]. Another study observed a decrease in cost per defined daily dose after medicine price negotiation and reimbursement [24]. Our study adds to the evidence by exploring the impact of price negotiations and the subsequent expansion of BMI benefits to incorporate 15 TAMs into NRDL in China in 2017 using individual-level data. The rise in prescriptions of price-negotiated TAMs may be mainly driven by increased affordability of the medicines for the patients due to price decreases after negotiation and the partial reimbursement of BMI fund.

Studies on the financial impact of health insurance expansion produced varying results, although most showed an increase in drug prescriptions after expansion. In the United States, a review concluded that Medicare Part D enrolees have increased drug utilisation while experiencing reduced OOP costs [35]. By contrast, a 19.2% annualised growth rate of oncology drug expenditures was observed in Chile from 2012-2013 to 2016-2017, with a gradual increase in OOP costs after expanded coverage [36]. The impact of insurance coverage expanded in Korea was concluded as co-payments decreased, but overall costs increased due to increased utilisation [37]. However, we found that, without a significant increase in OOP costs, the NRDL expansion to an additional 15 price-negotiated TAMs promoted their use among some patients without increasing their financial burden attributable to medical costs. This may be due to patients now being able to access TAMs which had a high cost prior to the NRDL expansion. Consequently, there may be an increase in direct medical costs but no noticeable change in OOP expenses because of insurance reimbursement. Presumably, alleviated financial burden can benefit patients’ clinical outcomes through increased treatment access and adherence. Other countries could use this to their advantage in drug price negotiations by being well-informed about financial hardship and patient harm [38].

In China, the level of economic development varies among the eastern, central, and western regions, as does the allocation of health resources and services [39]. One study concluded that disparities in cancer treatment were mainly driven by differences in the financial capability of cancer patients [40], which is also likely exacerbated by regional differences in economic development and allocation of medical resources [41]. Additionally, health insurance schemes may play an important role in health inequities [42,43]. The aOR values in our analysis decreased after policy implementation, indicating that the 2017 NRDL expansion promoted equity in TAM use among patients of different socioeconomic statuses. Nevertheless, the current medical insurance policy alone cannot address these disparities and requires sustained attention of policymakers. Moreover, considering that the Chinese disposable income per capita in 2017 was US$3848, the current negotiated medicine prices remain unaffordable for many patients [31]. Limited accessibility of TAMs may compromise patients’ clinical outcomes, which should be considered in further health care system reforms. Studies have found that medical insurance expansion was associated with substantial improvements in mental health and access to care for chronic conditions [44]. Future research could also focus on the impact of NRDL expansion policy on patients’ clinical outcomes.

Our study has some limitations. First, the database only had data from 2017, so we were unable to observe the long-term impact of price negotiation on the utilisation of TAMs. Second, it did not include uninsured patients, so the OOP cost could be underestimated. However, we expect that this did not significantly influence our findings, as the sample already covered over 95% of the national population in 2017 [45]. Third, we cannot identify patients’ specific medical needs due insufficient patient diagnoses information (e.g. gene tests) in the database. Thus, we cannot determine which patients should have been treated with these price-negotiation TAMs. Finally, we cannot conduct further analyses due to the lack of socioeconomic information in the database, such as patients’ real income and occupation.

## CONCLUSIONS

China’s medicine price negotiation and reimbursement policy in 2017 improved the accessibility of TAMs to cancer beneficiaries, and reduced disparities in TAM prescriptions among patients with different health insurance schemes or residing in different regions. This could serve as a model for other health systems. Further studies should focus on the long-term impact of the policy and clinical outcomes among cancer patients.

## Additional material


Online Supplementary Document
