# Supplementary material for: The impact of government reimbursement negotiation on targeted anticancer medicines use and cost in China: A cohort study based on national health insurance data
Source: J Glob Health. 2023 Aug 11;13:04083. doi: 10.7189/jogh.13.04083 (PMC10420358; doi:10.7189/jogh.13.04083)
Supplement: Online Supplementary Document [file jogh-13-04083-s001.pdf]

## Supplementary Documents

Figure S1. Patient identification flowchart.

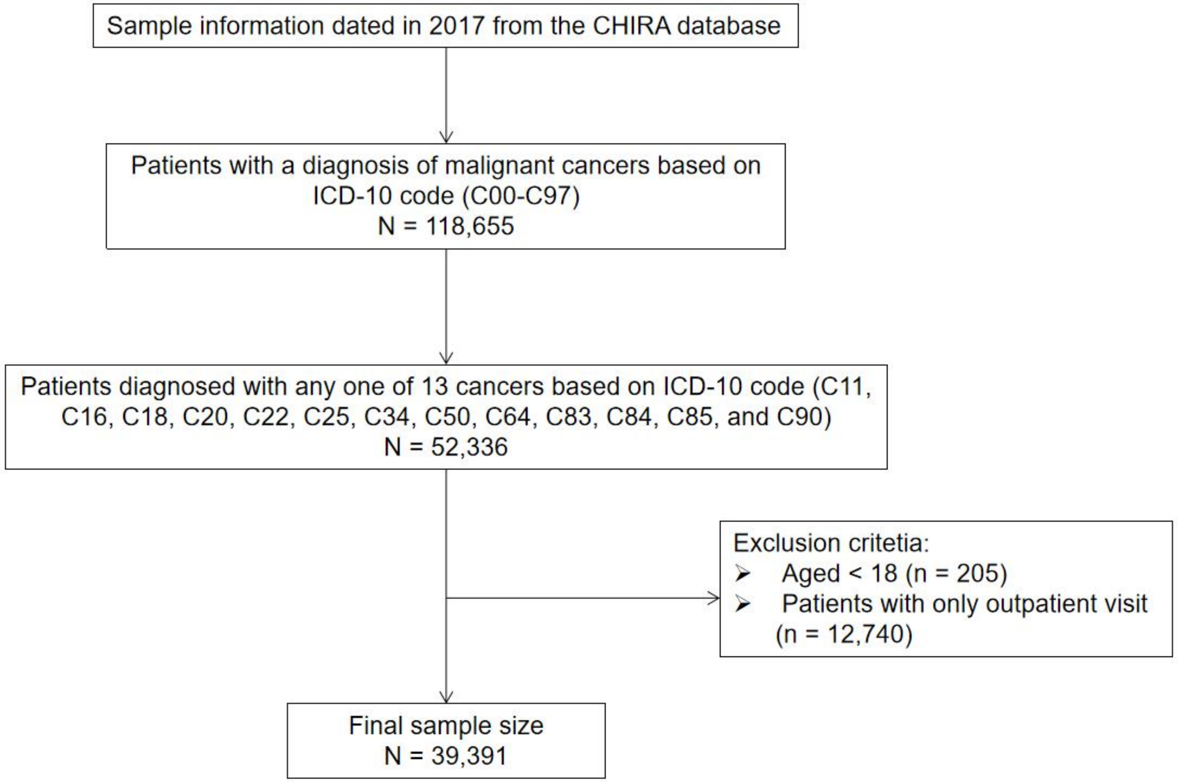


CHIRA – the China Health Insurance Association, ICD-10 – the International Classification of Diseases 10th Revision.

Table S1. Characteristics of sample targeted anticancer medications.

| No. | Generic Name | Indication | Target | Dosage Form | Negotiated price (2017 US $) |
|---|---|---|---|---|---|
| 1 | Trastuzumab | Breast cancer; Stomach cancer | ERBB2 | Injection | 1125.9 (440mg(20ml)/ vial) |
| 2 | Bevacizumab | Colorectal cancer; Lung cancer | VEGFA | Injection | 296 (100mg(4ml)/ vial) |
| 3 | Nimotuzumab | Nasopharynx cancer | EGFR | Injection | 251.9 (10ml: 50mg/vial) |
| 4 | Rituximab | Lymphoma | MS4A1 | Injection | 358.2 (100mg/10ml/vial)<br>1228.1 (500mg/50ml/vial) |
| 5 | Erlotinib | Lung cancer | EGFR | Oral | 28.9 (150mg/tablet)<br>21.2 (150mg/tablet) |
| 6 | Sorafenib | Liver cancer; Kidney cancer; Thyroid cancer | BRAF; FGFR1; FLT1; FLT3; FLT4; KDR; KIT; PDGFRB; RAF1; RET | Oral | 30.1 (0.2g/tablet) |
| 7 | Lapatinib | Breast cancer | EGFR; ERBB2 | Oral | 10.4 (250mg/tablet) |
| 8 | Apatinib | Stomach cancer | VEGFR-2 | Oral | 20.1 (250mg/tablet)<br>27.5 (375mg/tablet)<br>30.2 (425mg/tablet) |
| 9 | Bortezomib | Multiple myeloma; Lymphoma | PSMB1; PSMB2; PSMB5; PSMD1; PSMD2 | Injection | 906.1 (3.5mg/vial)<br>347.3 (1mg/vial) |
| 10 | Recombinant Human Endostatin | Lung cancer | Multi-target Antiangiogenesis | Injection | 93.3（15mg/2.4×10^5 U/3ml/vial） |

| No. | Generic Name | Indication | Target | Dosage Form | Negotiated price (2017 US $) |
|---|---|---|---|---|---|
| 11 | Chidamide | Lymphoma | HDAC | Oral | 57.0 (5mg/tablet) |
| 12 | Abiraterone | Prostate cancer | CYP17A1 | Oral | 21.5 (250mg/tablet) |
| 13 | Fulvestrant | Breast cancer | ESR1 | Injection | 355.6 (5ml: 0.25g/vial) |
| 14 | Everolimus | Brain cancer; Kidney cancer; Pancreatic cancer | MTOR | Oral | 21.9 (5mg/tablet)<br>12.9 (2.5mg/tablet) |
| 15 | Lenalidomide | Multiple myeloma | CRBN | Oral | 128.3 (5mg/capsule)<br>163.3 (5mg/capsule) |

MS4A – membrane-spanning 4-domains, subfamily A, ERBB2 – erb-b2 receptor tyrosine kinase 2, PSMB – proteasome subunit beta, PSMD – proteasome modulator, EGFR – epidermal growth factor receptor, BRAF – v-raf murine sarcoma viral oncogene homolog B, FGFR – fibroblast growth factor receptor, FLT – fms-like tyrosine, KDR – kinase insert domain receptor, KIT – KIT Proto-Oncogene, Receptor Tyrosine Kinase, PDGFRB – platelet-derived growth factor receptor beta, RAF – rapidly accelerated fibrosarcoma, RET – rearranged during transfection, VEGFA – vascular endothelial growth factor A, ESR – Estrogen Receptor, MTOR – mammalian/mechanistic targets of rapamycin, CRBN – Cereblon, HDAC – histone deacetylase, CYP17A1 – Cytochrome P450 Family 17 Subfamily A Member 1.
